# COVID-19 lockdowns and gestational diabetes risk: trimester-specific exposure and stratified outcomes

**DOI:** 10.1186/s12884-026-09446-x

**Published:** 2026-06-13

**Authors:** Omri Dominsky, Yariv Yogev, Liat Lerner-Geva

**Affiliations:** 1 Department of Obstetrics and Gynecology, Lis Hospital for Women’s Health, Sourasky University Medical Center (Ichilov), Tel Aviv, Israel; 2 Gray Faculty of Medical & Health Sciences, Tel Aviv University, Tel Aviv, Israel; 3 School of Public Health, Gray Faculty for Medical & Health Sciences, Tel Aviv University, Tel Aviv, Israel

**Keywords:** Gestational diabetes, COVID-19, Lockdown, Maternal health, Public-health preparedness, Health policy

## Abstract

**Background:**

The COVID-19 pandemic disrupted physical activity, nutrition, and stress, key determinants of gestational diabetes mellitus (GDM). We examined whether pandemic-period delivery and lockdown exposure were associated with GDM prevalence.

**Methods:**

We conducted a historical cohort study at a tertiary medical center including singleton deliveries ≥ 24 weeks during April 2018–October 2019 (pre-pandemic) and April 2020–October 2021 (pandemic). The pandemic period encompassed three national lockdowns. The primary outcome was GDM diagnosed using standardized two-step screening. Secondary outcomes included cesarean delivery and macrosomia. Multivariable logistic regression adjusted for maternal age, pre-pregnancy BMI, parity, chronic hypertension, and pre-gestational diabetes. Trimester-specific lockdown exposure was assessed.

**Results:**

Among 31,060 deliveries (15,190 pre-pandemic; 15,870 pandemic), GDM prevalence increased from 5.4% to 7.8% (*p* < 0.001). After adjustment, the association remained significant (adjusted OR 1.29, 95% CI 1.12–1.49). First-trimester lockdown exposure showed the strongest association (adjusted OR 1.6, 95% CI 1.3–2.0). Despite rising GDM, cesarean delivery rates and macrosomia remained stable, though cesarean for suspected macrosomia increased (5.7% to 8.1%, *p* < 0.001). Mean gestational weight gain did not differ between periods.

**Conclusions:**

Pandemic-period delivery was associated with increased GDM independent of gestational weight gain, potentially related to stress-mediated metabolic dysregulation, dietary quality changes, or reduced physical activity beyond caloric excess. First-trimester lockdown exposure showed the strongest association with GDM risk. These findings underscore the need for proactive interventions during public health emergencies, including digital health infrastructure, early pregnancy support, and integrated mental health care.

## Background

Gestational diabetes mellitus (GDM) is one of the most common medical complications of pregnancy, affecting an estimated 5–15% of pregnancies globally, with substantial variation by region, ethnicity, and diagnostic criteria [1]. GDM is associated with increased risk of hypertensive disorders of pregnancy, operative delivery, and neonatal complications [2], and is a powerful predictor of maternal type 2 diabetes and offspring metabolic dysfunction [1, 3, 4]. In local population-based data, the prevalence has been reported at approximately 4% [5].

The COVID-19 pandemic disrupted the behavioral and environmental determinants of maternal metabolism on an unprecedented scale. Strict national lockdowns implemented worldwide included school closures, work-from-home mandates, closure of gyms and recreational facilities, and restrictions on outdoor movement. These measures, while effective in limiting viral spread, were associated with substantial reductions in physical activity, weight gain, and adverse emotional states among adult populations [6, 7]. Pregnant women worldwide reported heightened anxiety, depressive symptoms, and reduced physical activity during the pandemic [8].

International evidence on the pandemic’s impact on GDM prevalence has been conflicting. While studies from regions with stringent social restrictions, such as northeast Italy and parts of China, have reported higher GDM rates among women pregnant during lockdowns, with some analyses suggesting vulnerability when lockdowns overlapped with early pregnancy [9, 10]. In contrast, a recent analysis from Shanghai reported stable or even reduced GDM rates during lockdown [11]. Beyond metabolic complications, emerging reports have also suggested that pandemic lockdowns may have influenced birth timing, with some studies reporting changes in rates of preterm birth and gestational length [11, 12].

These mixed findings raise important questions about how national policies, healthcare organization, and population behavior shape metabolic risk during pregnancy. Understanding lockdown impacts on GDM in a universal healthcare setting with standardized screening protocols provides insights relevant to diverse health systems worldwide, as most developed nations implemented similar restrictions while attempting to maintain prenatal care. The unique combination of universal health coverage, standardized GDM screening protocols applied uniformly across all healthcare providers, and stringent lockdown policies creates an informative context for examining how public health emergency measures affect maternal metabolic outcomes. Such findings have direct implications for pandemic preparedness planning across different healthcare systems.

The objective of this study was to evaluate whether GDM prevalence increased among women who delivered during the COVID-19 pandemic compared with a pre-pandemic cohort, and to examine whether lockdown exposure during specific trimesters of pregnancy was differentially associated with GDM risk. We hypothesized that pandemic-period delivery would be associated with higher GDM prevalence independent of traditional risk factors, and that first-trimester lockdown exposure would show the strongest association with GDM risk.

## Methods

### Study design and setting

We conducted a historical cohort study at tertiary referral center serving a diverse urban population within a universal healthcare system, with approximately 12,500 deliveries annually. We compared two delivery cohorts: a pre-pandemic period (April 2018 to October 2019) and a pandemic period (April 2020 to October 2021) spanning the main COVID-19 waves and national lockdowns. Deliveries between November 2019 and March 2020 were excluded to avoid misclassification during the pandemic’s early transition phase. The study protocol was approved by the Institutional review board with a waiver of informed consent for retrospective use of anonymized data.

### Study population

We included all women delivering a singleton live birth at or beyond 24 + 0 weeks’ gestation. Exclusion criteria were multiple gestation, stillbirth or termination of pregnancy, and missing data on key variables including pre-pregnancy BMI, gestational weight gain, GDM status, or birth weight. Gestational age was determined based on first-trimester ultrasound dating when available or last menstrual period otherwise, consistent with departmental practice.

### Exposure definition

The primary exposure was delivery during the pandemic period compared with the pre-pandemic period. For trimester-specific analyses (which were pre-specified secondary analyses), we classified maternal lockdown exposure by calculating the overlap between the estimated conception date and each of three national lockdowns. The dates of the three lockdown periods were: (1) March 15 to May 17, 2020 (9 weeks); (2) September 17 to October 18, 2020 (4 weeks); and (3) January 8 to February 11, 2021 (4 weeks). During these lockdowns, health and fitness clubs were closed at the national level, and during the first lockdown citizens were prohibited from venturing more than 100 m from their homes.

First trimester was defined as conception to 13 + 6 weeks, second trimester as 14 + 0 to 27 + 6 weeks, and third trimester as 28 + 0 weeks until delivery. A woman was considered exposed in each trimester, if any part of that trimester overlapped with an official national lockdown. Women could have been exposed in more than one trimester depending on conception date and gestational age at lockdown onset.

### Outcome definition

The primary outcome was GDM diagnosed according to national guidelines based on a two-step screening strategy uniformly applied across all healthcare providers [13]. All women without known pre-gestational diabetes were offered a 50 g glucose challenge test (GCT) at 24–28 weeks’ gestation. Women with GCT values ≥ 200 mg/dL were diagnosed with GDM without further testing. Those with GCT values between 140 and 199 mg/dL underwent a 100 g, 3-hour OGTT. GDM was diagnosed if at least one OGTT value met or exceeded Carpenter–Coustan thresholds: fasting ≥ 95 mg/dL, 1-hour ≥ 180 mg/dL, 2-hour ≥ 155 mg/dL, or 3-hour ≥ 140 mg/dL [14]. Women with GDM were classified as A1 (diet-controlled) or A2 (requiring insulin therapy) based on clinical management. Pre-gestational diabetes (type 1 or type 2) was identified from medical records. GDM diagnosis was abstracted from the electronic medical record based on laboratory-confirmed blood glucose testing meeting the diagnostic criteria described above. All diagnoses were based on documented glucose challenge test results and, when indicated, oral glucose tolerance test results, rather than patient self-report. Classification of GDM as diet-controlled (A1) versus insulin-requiring (A2) was based on documented clinical management during prenatal care.

Secondary outcomes included overall cesarean delivery rate, cesarean delivery for suspected macrosomia, actual macrosomia (birth weight > 4,000 g) instrumental delivery, preterm delivery (< 37 + 0 weeks), hypertensive disorders of pregnancy, obstetric anal sphincter injury (3rd or 4th degree perineal tears), and small-for-gestational-age birth, defined as birth weight below the 10th percentile for gestational age and sex based on local reference curves [15]. Postpartum hemorrhage was defined as estimated blood loss exceeding 500 mL for vaginal deliveries or 1,000 mL for cesarean deliveries.

### Covariates

The following variables were abstracted from the hospital’s computerized perinatal database: maternal age, pre-pregnancy BMI, parity, chronic hypertension, pre-gestational diabetes, and gestational weight gain. Gestational weight gain was calculated as the difference between weight at delivery and pre-pregnancy weight. Weight at delivery was measured using calibrated hospital scales during the admission process. Pre-pregnancy weight was abstracted from prenatal care records documented at the first prenatal visit. Neonatal data included birth weight and Apgar scores.

### Statistical analysis

Baseline characteristics, Obstetric and neonatal outcomes were compared between periods using chi-square tests for categorical variables and t-tests or Mann–Whitney tests for continuous variables. GDM prevalence and subtypes (A1 and A2) were calculated for each period.

Multivariable logistic regression models estimated the association between pandemic-period delivery and GDM, adjusted for maternal age, pre-pregnancy BMI, parity, chronic hypertension, and pre-gestational diabetes, with results expressed as adjusted odds ratios (aORs) with 95% CIs. To assess trimester-specific lockdown effects, we constructed separate models with mutually adjusted binary indicators for first-, second-trimester exposure, using the pre-pandemic cohort as reference. Third-trimester exposure was not assessed because GDM diagnosis occurs at 24–28 weeks’ gestation. Statistical significance was set at *p* < 0.05. Analyses were performed using IBM SPSS Statistics version 26.0 (IBM Corp., Armonk, NY).

## Results

Of 37,424 eligible deliveries, 6,364 (17%) were excluded due to missing data, yielding 31,060 deliveries for analysis: 15,870 (51%) during the pandemic and 15,190 (49%) during the pre-pandemic. Baseline maternal characteristics were largely similar between the two periods (Table 1). Maternal age was comparable (mean 33 ± 4.9 years pre-pandemic vs. 33 ± 4.7 years pandemic, *p* = 0.692), as were pre-pregnancy BMI (22.8 ± 4.2 vs. 22.8 ± 4.2 kg/m², *p* = 0.133). The prevalence of obesity (BMI ≥ 30) was similar between periods (6.5% vs. 6.9%, *p* = 0.203). The pandemic group had a modestly higher proportion of nulliparous women (45.7% vs. 44.1%, *p* = 0.003) and a lower prevalence of chronic hypertension (0.5% vs. 0.7%, *p* = 0.006). Pre-gestational diabetes prevalence did not differ between periods (0.4% vs. 0.3%, *p* = 0.632).

**Table 1:** Baseline Characteristics and pregnancy outcomes by study period

Variable	Pre-pandemic (*n* = 15,190)	Pandemic (*n* = 15,870)	*P* value	OR (95% CI)
Baseline maternal characteristics				
Maternal age, years (mean ± SD)	33 ± 4.9	33 ± 4.7	0.692	—
Pre-pregnancy BMI, kg/m² (mean ± SD)	22.8 ± 4.2	22.8 ± 4.2	0.133	—
BMI ≥ 30 (obesity), n (%)	988 (6.5)	1,090 (6.9)	0.203	1.06 (0.97–1.16)
Gravidity (mean ± SD)	2.2 ± 1.6	2.4 ± 1.6	0.001	—
Parity (mean ± SD)	0.94 ± 1.2	0.89 ± 1.1	0.001	—
Nulliparous, n (%)	6,693 (44.1)	7,259 (45.7)	0.003	1.07 (1.02–1.11)
Pre-gestational diabetes, n (%)	56 (0.4)	53 (0.3)	0.632	0.90 (0.61–1.33)
Chronic hypertension, n (%)	109 (0.7)	76 (0.5)	0.006	0.67 (0.49–0.90)
Gestational diabetes				
Overall GDM, n (%)	817 (5.4)	1,244 (7.8)	< 0.001	1.49 (1.36–1.63)
Diet-controlled GDM (A1), n (%)	654 (4.3)	1,039 (6.5)	< 0.001	1.55 (1.4–1.7)
Insulin-treated GDM (A2), n (%)	163 (1.1)	205 (1.3)	0.054	1.2 (0.99–1.5)
Maternal outcomes				
Gestational weight gain, kg (mean ± SD)	12.6 ± 5.2	12.6 ± 5.3	0.800	—
Gestational hypertension or preeclampsia, n (%)	153 (1.0)	150 (0.9)	0.603	0.93 (0.74–1.1)
Preterm birth < 37 weeks, n (%)	690 (4.5)	587 (3.7)	< 0.001	0.8 (0.7–0.9)
Post-term > 41 weeks, n (%)	1,975 (13.0)	1,971 (12.4)	0.125	0.94 (0.88–1.01)
Spontaneous onset of labor, n (%)	10,031 (67.6)	10,305 (65.8)	< 0.001	0.92 (0.87–0.96)
Labor induction, n (%)	2,327 (15.7)	2,549 (16.3)	0.164	1.04 (0.98–1.1)
Obstetric Anal Sphincter Injury, n (%)	55 (0.5%)	58 (0.5%)	1.000	1 (0.69–1.46)
Postpartum hemorrhage, n (%)	289 (1.9)	287 (1.8)	0.556	0.95 (0.8–1.12)
Mode of delivery				
Vaginal deliveries, n (%)	12,025 (79.2%)	12,565 (79.2%)	0.989	1 (0.94–1.05)
Instrumental delivery, n (%)	1269 (8.4%)	1522 (9.6%)	0.001	1.16 (1.07–1.25)
Overall cesarean delivery, n (%)	3,165 (20.8)	3,305 (20.8)	0.989	0.99 (0.94–1.05)
Elective cesarean, n (%)	1,708 (56.1)	1,930 (59.5)	0.006	1.06 (1.01–1.12)
Cesarean for suspected macrosomia, n (%)	181 (5.7)	268 (8.1)	< 0.001	1.45 (1.19–1.76)
Neonatal outcomes				
Macrosomia > 4,000 g, n (%)	661 (4.4)	674 (4.2)	0.654	0.97 (0.87–1.08)
Large-for-gestational-age > 90th percentile, n (%)	1,045 (6.9)	1,110 (7.0)	0.704	1.01 (0.93–1.1)
Small-for-gestational-age < 10th percentile, n (%)	266 (1.8)	496 (3.1)	< 0.001	1.8 (1.5–2.1)
Low birth weight < 2,500 g, n (%)	715 (4.7)	687 (4.3)	0.113	0.91 (0.82–1.02)
Shoulder dystocia, n (%)	51 (0.4)	50 (0.4)	0.654	0.9 (0.6–1.2)
Apgar score < 7 at 5 min, n (%)	37 (0.2)	48 (0.3)	0.330	1.24 (0.8–1.9)

*BMI* Body mass index, *GDM* Gestational diabetes mellitus, *OR* Odds ratio, *CI* Confidence interval, *SD* Standard deviation

### Prevalence of gestational diabetes

Overall, 2,061 of 31,060 women (6.6%) were diagnosed with GDM. GDM prevalence increased from 5.4% (817/15,190) in the pre-pandemic period to 7.8% (1,244/15,870) during the pandemic period (*p* < 0.001), corresponding to a 44% relative increase (Table 1). This rise was driven primarily by diet-controlled GDM (A1), which increased from 4.3% to 6.5% (*p* < 0.001). Insulin-treated GDM (A2) increased more modestly, from 1.1% to 1.3% (*p* = 0.054).

#### Maternal outcomes

Preterm birth before 37 weeks occurred in 4.5% of deliveries in the pre-pandemic period and 3.7% in the pandemic period (OR 0.8, 95% CI 0.7–0.9, *p* < 0.001). Post-term delivery (> 41 weeks) occurred in 13.0% of pre-pandemic deliveries and 12.4% of pandemic deliveries (*p* = 0.125). Spontaneous onset of labor occurred in 67.6% of pre-pandemic deliveries and 65.8% of pandemic deliveries (OR 0.92, 95% CI 0.87–0.96, *p* < 0.001). Labor induction rates were 15.7% in the pre-pandemic period and 16.3% in the pandemic period (*p* = 0.164). The prevalence of gestational hypertension or preeclampsia was 1.0% in the pre-pandemic period and 0.9% in the pandemic period (*p* = 0.603). Postpartum hemorrhage rates were 1.9% in the pre-pandemic period and 1.8% in the pandemic period (*p* = 0.556).

#### Mode of delivery

The overall cesarean delivery rate was 20.8% in both the pre-pandemic and pandemic periods (*p* = 0.989) (Table 1). Among cesarean deliveries, the proportion that were elective increased from 56.1% to 59.5% (OR 1.06, 95% CI 1.01–1.12, *p* = 0.006). Cesarean sections performed for suspected macrosomia increased from 5.7% to 8.1% (OR 1.45, 95% CI 1.19–1.76, *p* < 0.001). Mean gestational weight gain was 12.6 ± 5.2 kg in the pre-pandemic period and 12.6 ± 5.3 kg in the pandemic period (*p* = 0.800). Instrumental delivery rates were 8.4% in the pre-pandemic period and 9.6% in the pandemic period (OR 1.16, 95% CI 1.07–1.25, *p* < 0.001). Rates of obstetric anal sphincter injury were 0.5% in both periods (*p* = 1.0).

#### Neonatal outcomes

Macrosomia (> 4,000 g) occurred in 4.4% of pre-pandemic births and 4.2% of pandemic births (*p* = 0.654). Large-for-gestational-age birth (> 90th percentile) occurred in 6.9% of pre-pandemic births and 7.0% of pandemic births (*p* = 0.704). Small-for-gestational-age birth (< 10th percentile) occurred in 1.8% of pre-pandemic births and 3.1% of pandemic births (OR 1.8, 95% CI 1.5–2.1, *p* < 0.001). Low birth weight (< 2,500 g) occurred in 4.7% of pre-pandemic births and 4.3% of pandemic births (*p* = 0.113). Shoulder dystocia occurred in 0.4% of deliveries in both periods (*p* = 0.654). Apgar scores < 7 at 5 min occurred in 0.2% of pre-pandemic births and 0.3% of pandemic births (*p* = 0.330).

### Lockdown timing and GDM risk

Among women delivering during the pandemic period, 58.5% had first-trimester overlap with at least one national lockdown and 53.7% had second-trimester exposure.

GDM prevalence was 8.6% among women whose first trimester overlapped with a national lockdown (Table 2). In unadjusted analyses, first-trimester lockdown exposure was associated with an OR of 1.52 (95% CI 1.38–1.66, *p* < 0.001) compared to the pre-pandemic reference period. After adjustment for maternal age, pre-pregnancy BMI, parity, chronic hypertension, and pre-gestational diabetes, first-trimester exposure remained associated with GDM (aOR 1.6, 95% CI 1.3–2.0, *p* = 0.001). Second-trimester exposure was associated with GDM in both unadjusted (OR 1.25, 95% CI 1.13–1.38, *p* < 0.001) and adjusted analyses (aOR 1.2, 95% CI 1.1–1.3, *p* = 0.02).

**Table 2:** Gestational diabetes risk by trimester of lockdown exposure

Lockdown exposure	GDM prevalence, %	Unadjusted OR (95% CI)	*P* value	Adjusted OR (95% CI)*	*P* value
Pre-pandemic reference	5.4	1.0 (reference)	—	1.0 (reference)	—
First trimester	8.6	1.52 (1.38–1.66)	< 0.001	1.6 (1.3–2.0)	0.001
Second trimester	7.2	1.25 (1.13–1.38)	< 0.001	1.2 (1.1–1.3)	0.02

*Adjusted for maternal age, pre-pregnancy BMI, parity, chronic hypertension, and pre-gestational diabetes. Models are mutually adjusted for exposure in each trimester. *GDM* Gestational diabetes mellitus, *OR* Odds ratio, *CI* Confidence interval. Third-trimester lockdown exposure was not assessed because GDM diagnosis occurs at 24–28 weeks’ gestation”

### Multivariable analysis

In unadjusted analyses, delivery during the pandemic period was associated with higher odds of GDM (OR 1.49, 95% CI 1.36–1.63, *p* < 0.001). After adjustment for maternal age, pre-pregnancy BMI, parity, chronic hypertension, and pre-gestational diabetes, the association remained significant (aOR 1.29, 95% CI 1.12–1.49, *p* < 0.001) (Table 3). Maternal age and BMI were strong independent predictors, with an aOR of 1.04 per year of age (95% CI 1.04–1.06, *p* < 0.001) and 1.10 per BMI unit (95% CI 1.09–1.11, *p* < 0.001). Nulliparity was associated with higher GDM risk in adjusted models (aOR 1.21, 95% CI 1.10–1.34, *p* < 0.001), although the unadjusted association was null (OR 0.98, 95% CI 0.89–1.07, *p* = 0.721), suggesting confounding by age and parity distribution. Chronic hypertension showed a positive association with GDM in unadjusted analyses (OR 2.53, 95% CI 1.68–3.79, *p* < 0.001) but this was attenuated and no longer significant after adjustment (aOR 1.48, 95% CI 0.97–2.26, *p* = 0.070).

**Table 3:** Multivariable logistic regression analysis for gestational diabetes mellitus

Variable	Unadjusted OR (95% CI)	*P* value	Adjusted OR (95% CI)*	*P* value
Study period (pandemic vs. pre-pandemic)	1.49 (1.36–1.63)	< 0.001	1.29 (1.12–1.49)	< 0.001
Maternal age (per year)	1.05 (1.04–1.06)	< 0.001	1.04 (1.04–1.06)	< 0.001
Pre-pregnancy BMI (per kg/m²)	1.11 (1.10–1.12)	< 0.001	1.10 (1.09–1.11)	< 0.001
Nulliparity (yes vs. no)	0.98 (0.89–1.07)	0.721	1.21 (1.10–1.34)	< 0.001
Chronic hypertension (yes vs. no)	2.53 (1.68–3.79)	< 0.001	1.48 (0.97–2.26)	0.070

*Adjusted for all variables in the table. *OR* Odds ratio, *CI* Confidence interval, *BMI* Body mass index

### Outcomes stratified by GDM status

To examine whether outcomes differed by GDM status, we performed stratified analyses (Table 4).

**Table 4:** Maternal and neonatal outcomes stratified by GDM status

Variable	Pre-pandemic	Pandemic	*P* value
NON-GDM WOMEN (*n* = 28,999)	*n* = 14,373	*n* = 14,626	
Gestational weight gain, kg, mean ± SD	12.75 ± 5.17	12.79 ± 5.26	0.598
Newborn birth weight, g, mean ± SD	3246.65 ± 456.4	3259.58 ± 454.9	0.016
Macrosomia > 4000 g, n (%)	611 (4.3)	635 (4.3)	0.704

GDM WOMEN (*n* = 2,061)	*n* = 817	*n* = 1,**244**	
Gestational weight gain, kg, mean ± SD	10.79 ± 6.01	10.83 ± 5.94	0.878
Gestational age at delivery, weeks, mean ± SD	39.11 ± 1.37	39.25 ± 1.23	0.017
Newborn birth weight, g, mean ± SD	3330 ± 432	3312 ± 471	0.555
Macrosomia > 4000 g, n (%)	50 (6.1)	39 (3.1)	0.002

*GDM* Gestational diabetes mellitus, *SD* Standard deviation

Among women with GDM, mean gestational weight gain was 10.79 ± 6.01 kg in the pre-pandemic period and 10.83 ± 5.94 kg in the pandemic period (*P* = 0.878). Among women without GDM, mean gestational weight gain was 12.75 ± 5.17 kg in the pre-pandemic period and 12.79 ± 5.26 kg in the pandemic period (*P* = 0.598).

Mean birth weight among infants born to women with GDM was 3330 ± 432 g in the pre-pandemic period and 3312 ± 471 g in the pandemic period (*P* = 0.555). Among infants born to women without GDM, mean birth weight was 3246.65 ± 456.4 g in the pre-pandemic period and 3259.58 ± 454.9 g in the pandemic period (*P* = 0.016).

Macrosomia rates (> 4000 g) in the GDM subgroup were 6.1% (50/817) in the pre-pandemic period and 3.1% (39/1,244) in the pandemic period (*P* = 0.002). Mean gestational age at delivery among women with GDM was 39.11 ± 1.37 weeks in the pre-pandemic period and 39.25 ± 1.23 weeks in the pandemic period (*P* = 0.017). Macrosomia rates in the non-GDM population were 4.3% (611/14,373) in the pre-pandemic period and 4.3% (635/14,626) in the pandemic period (*P* = 0.704).

## Discussion

### Principal findings

In this large cohort from a major tertiary maternity center, delivery during the COVID-19 pandemic was associated with a 44% increase in GDM prevalence compared with the pre-pandemic period. This association persisted after adjusting for maternal age, BMI, parity, and chronic conditions (aOR 1.29, 95% CI 1.12–1.49). First-trimester lockdown exposure showed the strongest association with GDM (aOR 1.6, 95% CI 1.3–2.0), with the magnitude of association greater than that observed for second-trimester exposure.

### Comparison with other studies

Our findings align with reports from Italy and China showing higher GDM during lockdowns, particularly with early-pregnancy exposure [9, 10]. In contrast, data from Shanghai described reduced GDM prevalence, possibly reflecting reduced ambient pollution, altered healthcare-seeking, or different screening strategies [11]. The 44% increase we observed exceeds the prevalence reported in earlier population-based data from similar populations⁵ and cannot be explained by demographic shifts alone, suggesting that strict lockdown policies combined with universal screening created conditions that amplified pandemic-related metabolic disruption.

These findings have implications beyond the specific study setting. The weight gain paradox, rising GDM despite stable gestational weight gain, challenges simplistic assumptions about pandemic-related metabolic risk and suggests that lockdown impacts operated through multiple pathways. This pattern may be relevant to other healthcare systems that maintained prenatal screening during lockdowns, as most developed nations implemented similar restrictions while attempting to preserve maternal health services.

### Novel contributions

Our study makes several unique contributions to the pandemic-era obstetric literature. First, we provide trimester-specific lockdown exposure analysis demonstrating differential vulnerability by pregnancy timing, which has important implications for targeting interventions during future public health emergencies. Second, our GDM-stratified outcome data demonstrate that macrosomia rates decreased within the GDM subgroup despite later delivery timing, suggesting improved glycemic control during the pandemic despite increased GDM prevalence. This paradoxical improvement in GDM outcomes provides reassurance that pandemic-associated increases in GDM diagnosis were accompanied by successful clinical adaptation. Third, we demonstrate that GDM increased despite stable gestational weight gain in both GDM and non-GDM subgroups, providing evidence that pandemic-related metabolic disruption operated through pathways independent of total caloric excess. Fourth, our study benefits from universal healthcare coverage with standardized GDM screening protocols applied uniformly across all providers, minimizing selection bias and screening heterogeneity.

### Baseline GDM prevalence in context

The pre-pandemic GDM prevalence of 5.4% in our cohort is modestly higher than the local population-based average of approximately 4% reported in similar settings⁵. This difference may reflect our institution’s role as a tertiary referral center serving a diverse urban population, including women referred for high-risk conditions. However, our dataset was assembled specifically for this study and does not include deliveries before April 2018. We are therefore unable to determine institutional GDM prevalence in the years preceding the study period using the same cohort definition and diagnostic ascertainment, and the stability and representativeness of our pre-pandemic baseline over time cannot be confirmed. This should be considered when interpreting the magnitude of the observed pandemic-period increase.

### The weight gain paradox and outcomes in the GDM subgroup

The stratified analyses by GDM status provide critical insights into the mechanisms underlying our findings. First, the stable gestational weight gain within both GDM and non-GDM groups across study periods (12.75–12.79 kg for non-GDM women and 10.79–10.83 kg for GDM women) confirms that the 44% increase in GDM prevalence cannot be attributed to changes in overall weight gain patterns. Women with GDM consistently gained approximately 2 kg less than women without GDM in both periods, likely reflecting dietary counseling and glucose management following diagnosis.

Second, macrosomia rates among women with GDM decreased during the pandemic period (6.1% to 3.1%, *P* = 0.002) despite delivery at a slightly later gestational age (39.11 to 39.25 weeks, *P* = 0.017), ruling out earlier induction as an explanation. This contrasts with stable macrosomia rates in the non-GDM population (4.3% in both periods). Potential explanations include improved glycemic control through increased home monitoring due to reduced clinic visits, enhanced telemedicine support, heightened clinical vigilance during the pandemic, or increased patient engagement with self-management strategies during lockdowns when other activities were restricted. An alternative explanation is that screening practices or diagnostic thresholds may have shifted during the pandemic, resulting in diagnosis of women with milder hyperglycemia who were more responsive to lifestyle interventions.

Third, birth weights remained stable across both GDM and non-GDM groups when comparing pre-pandemic and pandemic periods. The modest 12.9 g increase in mean birth weight among infants born to non-GDM mothers, while statistically significant given the large sample size, is clinically insignificant. Together, these findings demonstrate that the pandemic-associated increase in GDM occurred through pathways independent of gestational weight gain, consistent with stress-mediated metabolic dysregulation, dietary quality changes, or reduced physical activity affecting glucose metabolism without substantial effects on overall weight gain.

### Potential mechanisms

Lockdown measures globally reduced physical activity and increased weight gain in general populations [6, 7]. Given physical activity’s central role in maintaining insulin sensitivity, abrupt declines may have worsened gestational insulin resistance, particularly among women already at intermediate metabolic risk. Pandemic-related psychological stress, which is associated with hypothalamic-pituitary-adrenal axis dysregulation and heightened inflammatory tone, may have further impaired glucose metabolism [8]. Dietary changes, including greater reliance on energy-dense foods and irregular meal timing, could have increased glycemic excursions, especially during the early adaptive phase of pregnancy.

Importantly, gestational weight gain remained similar between periods in our study, consistent with the hypothesis that stress-induced metabolic disruption, reduced physical activity independent of weight gain, and dietary quality changes (rather than quantity alone) may have increased GDM risk beyond what total weight gain would predict. First-trimester lockdown exposure showed the strongest GDM association, consistent with early pregnancy representing a period when disruptions in energy balance, physical activity, or stress physiology may set metabolic patterns that manifest as GDM at routine screening in the late second trimester. Similar principles of “metabolic imprinting” have been described in offspring of mothers with GDM, who exhibit increased rates of obesity, glucose intolerance, and cardiovascular risk factors across the life course [1, 3, 4]. Our findings are consistent with the possibility that analogous mechanisms may apply to the maternal metabolic response to early pregnancy environments.

The relationship between pandemic-related stress and pregnancy outcomes is complex and multifactorial. While we hypothesize that psychological stress may have contributed to increased GDM through metabolic pathways, we acknowledge that preterm births decreased during the pandemic period in our cohort and internationally, potentially reflecting reduced iatrogenic preterm deliveries, decreased occupational physical demands with work-from-home policies, and changes in environmental exposures. The differential effects of pandemic conditions on various pregnancy outcomes underscore the complexity of these relationships and the limitations of attributing outcomes to single mechanisms without direct measurement of behavioral and physiological factors.

### Long-term implications for mothers and children

Women with GDM face markedly increased risk of type 2 diabetes and cardiovascular disease [4, 16, 17]. Population-based data show higher cardiovascular events in women with GDM more than a decade after pregnancy [17]. Offspring exposed to GDM have increased risk of obesity, glucose intolerance, and hypertension [1, 3, 4, 18, 19]. The pandemic-related GDM increase thus signals a future rise in diabetes and cardiovascular burden unless prevention strategies are implemented.

#### Obstetrical outcomes

Despite higher GDM prevalence, overall cesarean rates remained stable, though cesarean for suspected macrosomia increased without corresponding changes in actual macrosomia. This may reflect heightened concern about shoulder dystocia and limitations of fetal weight estimation. Small-for-gestational-age births increased while preterm births decreased in our cohort. International findings on preterm birth during lockdowns have been inconsistent, with some studies reporting increases and others decreases [11, 12]. Instrumental delivery increased modestly without increasing obstetric anal sphincter injury, suggesting clinical adaptation without compromising maternal outcomes. We also observed a decrease in post-term deliveries during the pandemic period, although this did not reach statistical significance (13.0% versus 12.4%, *P* = 0.125). While no firm conclusions can be drawn, this pattern may reflect changes in obstetric practice during the pandemic, such as a lower threshold for scheduled delivery to limit prolonged or repeated hospital attendance, and should be interpreted with caution given the absence of statistical significance.

### Strengths and limitations

Strengths include large sample size, standardized GDM screening, comprehensive perinatal database, and trimester-specific lockdown exposure classification within a universal healthcare setting. The pandemic cohort was larger than the pre-pandemic cohort (15,870 versus 15,190 deliveries) despite a 2.6% national decline in birth rates during 2020. The reasons for this cannot be determined from our data. One possibility is increased referrals to our tertiary center if smaller hospitals reduced obstetric services during the pandemic, although we have no data to confirm this. Another possibility is that the completeness of electronic medical record documentation improved during the pandemic period, resulting in fewer exclusions for missing data. Importantly, if documentation completeness did improve during the pandemic, this could have led to more complete ascertainment of GDM cases and may therefore have contributed to the observed increase in GDM prevalence. We are unable to exclude this possibility with the available data, and it should be considered when interpreting the magnitude of the observed association. Limitations include single-center design limiting generalizability, 17% missing data (though sensitivity analyses suggest minimal bias), lack of individual-level data on diet, physical activity, and psychological stress, absence of socioeconomic status indicators, lack of data on COVID-19 infection status, potential PPH underestimation due to clinical estimation methods, inability to definitively quantify GDM screening uptake rates in each period, and inability to fully exclude subtle screening changes or healthcare utilization patterns during the pandemic.

### Public health policy implications

Our findings reveal critical vulnerabilities in maternal healthcare systems during public health emergencies, with implications for pandemic preparedness worldwide. Despite maintained universal screening protocols, GDM prevalence increased substantially, suggesting that reactive clinical approaches are insufficient during societal disruptions.

Four priorities emerge for public health policy. First, healthcare systems should develop digital infrastructure enabling remote glucose monitoring and virtual prenatal consultations to maintain care continuity during movement restrictions. Second, given that first-trimester lockdown exposure showed the strongest association with GDM, early pregnancy preventive interventions addressing nutrition, physical activity, and stress management should be integrated into routine care and maintained during emergencies. Third, mental health screening and support should be embedded within prenatal services, recognizing the metabolic consequences of psychological stress. Fourth, maternal metabolic care should be explicitly designated as essential in emergency preparedness frameworks, with pre-positioned supplies and coordinated response protocols.

These reforms strengthen both routine maternal care and screening in addition to pandemic resilience, with potential cost savings from prevented diabetes and cardiovascular complications for mothers and offspring. The findings underscore the need for proactive, rather than reactive, approaches to protecting maternal metabolic health during public health crises.

## Conclusion

COVID-19 lockdowns were associated with a 44% increase in GDM prevalence, with first-trimester exposure showing the strongest association. These findings demonstrate how societal disruptions can profoundly affect maternal metabolism and highlight the need for adaptive healthcare systems during public health emergencies. Strengthening digital infrastructure, early pregnancy preventive interventions, and integrated mental health support, would better protect maternal metabolic health during future crises while improving routine prenatal care.

## Data Availability

The datasets generated and analyzed during the current study are not publicly available due to patient privacy regulations and institutional policies protecting confidential medical records but are available from the corresponding author on reasonable request with appropriate ethical approval and data sharing agreements.

## Abbreviations

GDM: Gestational diabetes mellitus
COVID-19: Coronavirus disease 2019
BMI: Body mass index
aOR: Adjusted odds ratio
CI: Confidence interval
OR: Odds ratio
GCT: Glucose challenge test
OGTT: Oral glucose tolerance test
CS: Cesarean section
SGA: Small for gestational age
LGA: Large for gestational age
IRB: Institutional review board
